# Barriers to Cancer Care Among People Experiencing Homelessness in Chicago

**DOI:** 10.7759/cureus.110465

**Published:** 2026-06-08

**Authors:** Ismihan A Uddin, Suleiman Mohiuddin, Majid M Mohiuddin

**Affiliations:** 1 Oncology, Midwestern University Chicago College of Osteopathic Medicine, Downers Grove, USA; 2 Oncology, Chi-Care Medical, Chicago, USA; 3 Radiation Oncology, Wake Forest School of Medicine, Winston-Salem, USA; 4 Radiation Oncology, Radiation Oncology Consultants, Park Ridge, USA

**Keywords:** cancer disparities, cancer screening access, chicago urban health, homelessness, mobile medical clinics

## Abstract

People experiencing homelessness (PEH) face profound cancer disparities, with frequent late-stage diagnosis, lower treatment completion rates than housed populations, and up to twofold higher cancer mortality. In Chicago, where homelessness is shaped by structural racism, economic disinvestment, and healthcare resource deserts, PEH carries a concentrated burden of cancer risk. Factors including high tobacco and alcohol use, chronic viral infections (hepatitis C virus (HCV), hepatitis B virus (HBV), HIV, and human papillomavirus (HPV)), severe dental disease, malnutrition, and untreated mental illness and substance use disorders contribute to the overrepresentation of specific malignancies, most notably lung, head and neck, cervical, liver, colorectal, and breast cancers, which are often missed early due to limited access to preventive care and screening. This review highlights critical gaps across the cancer care continuum (the comprehensive framework describing cancer prevention, detection, diagnosis, treatment, and survivorship) and underscores Chicago’s urgent need for targeted, low-barrier interventions. Mobile medical clinics represent a scalable and community-centered solution to deliver cancer risk assessment, screening, linkage to care, and patient navigation directly to PEH, offering a promising strategy to improve early detection, reduce cancer mortality, and advance cancer equity in Chicago.

## Introduction and background

Homelessness in Chicago is a multifaceted social condition defined at the federal level by the U.S. Department of Housing and Urban Development (HUD) as lacking a fixed, regular, and adequate nighttime residence, including individuals living in shelters, transitional housing, and places not meant for human habitation [1]. About 2.3-3.5 million people experience homelessness in the United States every year [2,3]. However, other local advocacy organizations, such as the Chicago Coalition for the Homeless (CCH), adopt a broader definition that also includes people who are temporarily staying with friends or family, capturing a more comprehensive picture of housing instability in urban contexts like Chicago. In Chicago alone, recent estimates suggest that 68-76000 individuals experience homelessness over the course of a year when doubled-up situations are included, with a point-in-time (PIT) count (standardized “snapshot” count of people experiencing homelessness (PEH) on a single night in late January) of over 18000 people without stable housing in 2024 [2]. The median age has risen over the last decade from 37 to 46 years, with an average age of 50 years, meaning many PEH fall within recommended cancer screening age groups [2].

The scale of homelessness in Chicago reflects both structural inequities and economic pressures. In 2024, 16.6% of Chicagoans were below the federal poverty level, compared to the national average of 12.1% [4]. People of color, particularly Black and migrant Hispanic Chicagoans, are disproportionately affected, and individuals experiencing homelessness range across age groups, including unaccompanied youth and families with children [5]. Roughly one-third of PEH are <18 years old, and 53% are Black in Chicago [2]. These demographic realities underscore the intersecting burdens of race, income, and housing stability that shape health outcomes in Chicago’s most vulnerable communities.

Cancer is a leading cause of mortality in the United States, accounting for approximately one in six deaths and remaining the second leading cause of death overall behind heart disease [6]. With hundreds of thousands of Americans expected to die from cancer annually and persistent disparities in cancer incidence and outcomes, access to early detection and effective treatment is a cornerstone of public health [7]. For PEH, these challenges are amplified: research indicates that PEH have higher cancer mortality rates, poorer access to preventive care, lower screening participation, and often present with more advanced disease stages and worse survival than housed populations [8]. Of cancer patients who died in 2018, an estimated 22% could have lived if they had equal access, quality of care, and treatment equivalent to those of college-educated persons [9]. Unhoused individuals in the Chicago area die an average of 20 years younger (age 56.3 years) than the general population (age 74.2 years) [10].

The compounded effect of unstable housing, limited access to healthcare, and social marginalization means that PEH in Chicago are at heightened risk across the entire cancer continuum, from prevention and early detection through treatment and survivorship. Despite increasing awareness of these inequities, targeted interventions in urban settings remain limited, making Chicago both a critical need area and an opportunity for innovative, community-centered models of care. By situating cancer burden within the lived realities of homelessness in Chicago, this article highlights why targeted intervention, such as mobile medical services, is necessary to bridge care gaps and improve outcomes for this underserved population [11].

Objectives/aims

This review aims to (1) characterize the cancer burden among PEH in Chicago and comparable urban settings, (2) identify barriers across the cancer care continuum, and (3) propose mobile and street medicine-based interventions to improve cancer screening, navigation, and treatment access.

Although prior reviews have described cancer disparities among PEH broadly, few have focused specifically on implementation barriers within Chicago’s urban safety-net ecosystems (interconnected networks of organizations, technologies, policies, and funding that collaborate to provide essential social, health, or financial support to vulnerable populations) or examined how street medicine and mobile care infrastructure could operationalize cancer prevention and navigation in a city such as Chicago [12-14]. For example, Dommaraju conducted a systematic review of sixteen studies of various interventions conducted mostly in urban settings, but none were Chicago-based [14]. Despite Chicago being a major city in the Midwest with a large population of PEH, only a couple of studies specific to Chicago were identified, with a few additional studies cited from Midwestern cities (Cleveland and Detroit) [15-19]. This review highlights this knowledge gap specific to Chicago while drawing on evidence from other urban centers.

Methods and review framework

This article is a narrative/scoping review rather than a formal systematic review or meta-analysis. The goal was to synthesize literature related to cancer disparities among PEH, with emphasis on barriers across the cancer continuum and implementation considerations for mobile oncology outreach in Chicago.

Literature searches were performed using PubMed, Google Scholar, and Scopus databases to identify relevant peer-reviewed studies, systematic reviews, public health reports, policy documents, and guideline statements related to homelessness, cancer disparities, cancer screening, mobile healthcare delivery, street medicine, and patient navigation.

Search terms included combinations of “homelessness,” “people experiencing homelessness,” “housing instability,” “cancer,” “oncology,” “cancer screening,” “mobile clinics,” “street medicine,” “patient navigation,” “health disparities,” “safety-net healthcare,” and “Chicago.” Boolean operators (“AND,” “OR”) were used to refine searches. Additional references were identified through citation review of relevant articles and inclusion of publicly available governmental and community health reports relevant to Chicago-specific healthcare infrastructure and homelessness trends. Titles and abstracts were screened independently by two reviewers (authors IU and MMM) to identify potentially eligible studies. The full texts of these potentially eligible studies were then retrieved and evaluated independently by the same two reviewers for final inclusion. Disagreements were resolved through consensus.

Included sources consisted primarily of observational studies, cohort studies, systematic reviews, public health literature, and healthcare policy reports addressing cancer epidemiology, risk factors, screening, treatment barriers, survivorship, and healthcare access among PEH. Non-English language sources were excluded. Selecting for AND “Chicago” revealed few studies, highlighting the knowledge gap. Therefore, sources were selected based on relevance to the cancer care continuum and applicability to underserved urban populations. Initially screened studies ranged from 1990 to 2026, with final review inclusion from 2000 to 2026. Identified organizations in Chicago were contacted via email and telephone from June to December 2025 for Table 1.

**Table 1:** Existing cancer screening and oncology infrastructure serving unhoused populations in Chicago IMAN, Inner-City Muslim Action Network; MSHC, Mile Square Health Center; FQHC, federally qualified health clinic; FIT, fecal immunochemical testing; ICN, Islamic Circle of North America; VA, Department of Veterans Affairs; UI, University of Illinois

Organization	Program type	Primary contact route	Phone	Website
Heartland Alliance Health	FQHC/homeless health	Main clinic/outreach line	773-275-2586	https://hahealth.org
MSHC	FQHC	Main line: Request care coordination or social work services.	312-996-2000	https://hospital.uillinois.edu/mile-square-health-center
Erie Family Health Center	FQHC	Front desk/scheduling (Humboldt Park)	312-666-3494	https://www.eriefamilyhealth.org
Lawndale Christian Health Center	FQHC	Appointment/referral coordination	872-588-3440	https://lawndale.org
IMAN Health Center	FQHC	Appointment/referral coordination	773-434-4626	https://imancentral.org/programs/iman-health-center/
Cook County Health Street Medicine	Street Medicine	System care coordination hub	312-864-0200	https://cookcountyhealth.org
UI Health Street Outreach	Street Medicine	Community outreach routing	312-355-5729 / 5730	https://hospital.uillinois.edu
The Night Ministry	Street Medicine	Health Outreach Team	773-256-7549	https://www.thenightministry.org
Cook County Health (Oncology)	Safety-net Oncology	Appointment and care coordination hub	312-864-0200	https://cookcountyhealth.org/service/oncology
UI Health Cancer Center	Safety-net Oncology	Cancer clinic scheduling	312-355-1625	https://hospital.uillinois.edu/cancer-center
Rush Community Programs	Safety-net Oncology	Community programs outreach line	708-660-2005	https://www.rush.edu/community
Sinai Community Programs	Safety-net Oncology	Community institute contact	773-257-6508	https://www.sinaichicago.org
Northwestern Community Programs	Safety-net Oncology	Center for Community Health	312-503-2580	https://www.nm.org/community
The Boulevard	Medical respite	Intake coordination	773-533-6013 ext. 231	https://www.blvd.org
Housing Forward	Medical respite/housing	Suburban Cook County Call Center	877-426-6515	https://www.housingforward.org
Franciscan Outreach	Medical respite/housing	Administrative main line	773-278-6724	https://www.franciscanoutreach.org
Salvation Army Medical Respite	Medical respite	Chicago Midwest Division main line	312-733-4801	https://centralusa.salvationarmy.org/usc/medical-respite
Mobile Mammography Units	Mobile screening	Mammography coordination	773-995-3093	https://www.chicagobreasthealth.org/mobile-mammography
Shelter-Based FIT Distribution	Mobile screening	Cook County Health coordination	312-864-0200	https://www.chicagocac.org(FIT/screening community programs)
Community Health Fairs	Mobile screening	CDPH main line	312-747-9884	https://www.chicago.gov/city/en/depts/cdph/supp_info/community-health-fairs.html
VA Chicago Healthcare System	Safety-net Oncology	Main system line	312-569-8387	https://www.va.gov/chicago-health-care
Shifa Clinic (ICN)	Community Clinic	Appointments-only clinic	630-364-4773	https://www.icnarelief.org/shifa-clinic
Malcolm X College Dental Clinic	Dental services	Dental clinic services	312-850-7060	https://www.ccc.edu/colleges/malcolm-x/pages/default.aspx
Chicago Dental Society	Dental referral resource	Main administrative office	312-836-7300	https://www.cds.org
Roseland Community Hospital Mobile Unit	Mobile screening	Hospital main line	773-995-3000	https://roselandhospital.org
PCC Walk-In Wellness Center (West Suburban Medical Center)	FQHC	Hospital switchboard	708-406-3929	https://www.pccwellness.org

**Figure 1:**
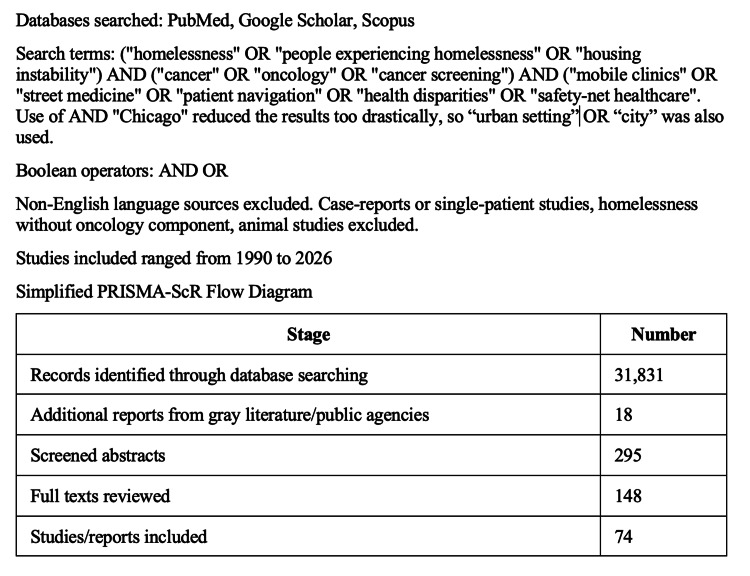
Literature search strategy with simplified PRISMA-ScR flow diagram PRISMA-ScR, Preferred Reporting Items for Systematic Reviews and Meta-Analyses Extension for Scoping Reviews

Because this manuscript was designed as a narrative review and policy-focused synthesis rather than a formal systematic review or meta-analysis, a Preferred Reporting Items for Systematic Reviews and Meta-Analyses Extension for Scoping Reviews (PRISMA)-guided study selection process and a formal risk-of-bias assessment tool were not applied. Priority was instead given to higher-quality evidence, including population-based studies, systematic reviews, and major public health publications. See Figure 1.

Epidemiology of cancer in people experiencing homelessness

Mortality and Incidence Trends

PEH bear a disproportionately high burden of cancer morbidity and mortality compared with the general population. Multiple cohort studies and population-based analyses demonstrate that cancer mortality among PEH is up to two times higher than that of housed individuals, even after adjustment for age and sex [12]. This excess mortality is driven not only by higher prevalence of risk factors, but also by systemic failures across the cancer care continuum, including delayed diagnosis, fragmented treatment, and poor continuity of care. Studies from urban centers in the United States, Canada, and Europe consistently show that cancer ranks among the leading causes of death for adults experiencing homelessness, often rivaling cardiovascular disease and overdose in older age groups [13]. In the Boston Health Care for the Homeless Program, cancer was the leading cause of death among unhoused individuals older than 45 years, and the second leading cause among those under 45 years [3]. Compared with the general population, PEH experience a four-fold higher prevalence and a two-fold higher mortality rate [13]. This suggests that homelessness itself may represent a high-risk condition warranting earlier screening and that general population screening guidelines may not fully apply to this population. Screening should begin at younger ages for PEH.

Additionally, late-stage presentation is a defining epidemiologic feature of cancer among PEH. Individuals experiencing homelessness are significantly more likely to be diagnosed at regional or metastatic stages, particularly for screen-detectable cancers such as breast, cervical, colorectal, and lung cancer [12].

Even after diagnosis, treatment completion rates are substantially lower among PEH. Evidence indicates that PEH are less likely to initiate definitive cancer therapy and far less likely to complete multimodal regimens involving surgery, chemotherapy, and/or radiation [13]. Interruptions due to housing instability, incarceration, psychiatric decompensation, and substance use are common. As a result, survival disparities persist even for cancers that are otherwise highly treatable when diagnosed early, underscoring that elevated mortality among PEH reflects systemic barriers rather than biologic differences alone.

As the financial gap in America increases, the number of PEH will likely continue to grow. Following the COVID-19 pandemic, there was a 33% increase in PEH in the United States by 2024 [14]. Shelters are reporting surges in occupancy, and the 2023 and 2024 PIT counts in Chicago also show increasing numbers, partly due to new arrivals such as migrants [2].

Risk Factor Burden

The elevated cancer burden among PEH is strongly influenced by a concentrated prevalence of behavioral, infectious, and environmental risk factors. Tobacco use is among the most well-documented contributors: smoking prevalence among PEH is estimated to range from 60% -80%, compared with approximately 12%-15% in the general U.S. population [20]. Heavy alcohol use is similarly overrepresented and contributes to cancers of the liver, esophagus, head and neck, and colorectum. These exposures act synergistically, particularly in squamous cell carcinomas of the upper aerodigestive tract.

Infectious carcinogenic exposures are also markedly elevated. PEH experience disproportionately high rates of chronic hepatitis B and C, leading to increased incidence of hepatocellular carcinoma (HCC) [21]. HIV prevalence among PEH is several-fold higher than in the general population, compounding immunosuppression-related cancer risks, including Kaposi sarcoma and non-Hodgkin lymphoma. Additionally, limited access to HPV vaccination and cervical cancer screening places unhoused individuals, particularly women and gender-diverse people, at heightened risk for HPV-associated malignancies, including cervical, anal, and oropharyngeal cancers [22].

Severe dental disease and chronic oral infections represent an underrecognized but critical cancer risk pathway in PEH. Poor oral hygiene, untreated periodontal disease, and chronic mucosal inflammation are widespread due to a lack of dental care access [23]. These conditions are associated with increased risk of oral and oropharyngeal cancers, which often present at more advanced stages. Malnutrition, micronutrient deficiencies, and inconsistent caloric intake further weaken immune surveillance and impair tissue repair mechanisms.

Mental illness and substance use disorders serve as compounding epidemiologic factors, intensifying cancer risk while simultaneously obstructing access to care. High rates of untreated depression, schizophrenia, bipolar disorder, and opioid or stimulant use disorder impair health-seeking behavior, reduce adherence to treatment, and increase the likelihood of care discontinuation. People worldwide with mental illness are more likely to die from cancer and less likely to undergo cancer screening compared with the general population [24]. These intersecting vulnerabilities create a cumulative cancer risk profile that far exceeds that of housed populations.

Structural and Environmental Contributors

Beyond individual risk factors, structural and environmental conditions play a decisive role in shaping cancer epidemiology among PEH in Chicago. Longstanding patterns of racial segregation and disinvestment, particularly on the city’s South and West Sides, have resulted in concentrated exposure to environmental carcinogens, including air pollution, industrial waste, and aging housing stock with lead and asbestos [25]. These neighborhoods, which disproportionately house Black and Latino residents, also experience higher rates of homelessness, compounding the issue.

Barriers to preventive care and early detection remain pervasive. PEH are significantly less likely to receive age-appropriate cancer screening, including mammography, Pap testing, colonoscopy, and low-dose CT for lung cancer. Insurance instability, lack of identification documents, transportation barriers, and fragmented referral pathways all contribute to missed opportunities for early diagnosis [12,26]. Even when screening is available through safety-net systems, follow-up of abnormal results is frequently incomplete.

Resource deserts further entrench these disparities. Many areas with high homelessness density lack accessible primary care clinics, oncology services, and dental care, forcing reliance on emergency departments for episodic care [27]. This episodic model is poorly suited to cancer prevention or management, reinforcing cycles of late diagnosis and poor outcomes. In this context, homelessness functions not merely as an individual risk factor but as a structural determinant of cancer epidemiology, necessitating innovative, community-based interventions capable of bridging gaps between marginalized populations and the healthcare system. Coughlin notes that to “address these social determinants, effective interventions are needed that account for the social and environmental contexts in which cancer patients and cancer survivors live and are treated” [28].

## Review

High burden and overrepresented cancers in the homeless populations

PEH are not uniformly affected by cancer; rather, specific malignancies are markedly overrepresented, reflecting concentrated exposure to behavioral risks, infectious agents, and structural barriers to prevention and care. These cancers are also disproportionately associated with late-stage diagnosis and poor survival, making them high-yield targets for intervention.

Lung Cancer

Lung cancer represents the highest incidence and highest mortality malignancy among PEH and is a leading contributor to excess cancer deaths in this population. Smoking prevalence among PEH is extraordinarily high, with estimates ranging from 60%-80%, often beginning at younger ages and continuing at higher intensity than in the general population. High rates of chronic obstructive pulmonary disease (COPD), asthma, and repeated respiratory infections further compound lung cancer risk and may obscure early symptoms [20]. Eighty-eight percent of lung cancers in PEH are thought to be smoking-attributable [3]. A Boston cohort study (2003-2008) found that lung cancer incidence and mortality rates were more than twofold higher among PEH compared to the general population, with a standardized mortality ratio of 1.88 for men and 1.61 for women [3,8].

Despite the availability of evidence-based screening via low-dose computed tomography (LDCT) for high-risk individuals, PEH have extremely limited access to lung cancer screening. Barriers include lack of primary care continuity, insurance instability, difficulty meeting eligibility documentation requirements, and limited follow-up capacity for abnormal results [29]. The INHALE trial in Boston demonstrated that with a patient navigation intervention, participants were 4.7-fold more likely to complete LDCT screening at six months compared to usual care, highlighting the extremely low baseline screening rates [8]. As a result, lung cancers among PEH are frequently diagnosed at advanced or metastatic stages, when curative options are limited. A San Francisco public hospital study found PEH were diagnosed with stage 4 disease 28% of the time compared to 22% of housed patients [30]. This pattern highlights lung cancer as both a major driver of mortality and a critical missed opportunity for early detection.

Head and Neck Cancers (Oral, Oropharyngeal, and Laryngeal)

Cancers of the oral cavity, pharynx, and larynx are substantially overrepresented among PEH and are closely linked to combined exposure to tobacco use, heavy alcohol consumption, and severe dental disease [31]. A London study of homeless populations found 52.4% brushed their teeth less than twice daily, 32.1% had experienced a toothache, and 80% were very or fairly concerned about their dental health [32]. Sixty percent of oropharyngeal cancers are thought to be smoking-attributable [3]. Chronic oral inflammation, untreated periodontal disease, poor dentition, and ill-fitting or absent dentures are widespread in homeless populations and contribute to carcinogenesis as well as delayed recognition of malignant lesions. The Edmonton study found that 54.7% of unhoused and underserved participants had clinical inflammatory changes in their oral mucosa, with a significant association between clinical inflammatory oral lesions and oral cancerous/precancerous lesions (p<0.001). The same study showed the risk of oral cancerous/precancerous lesions was 1.67 times higher in participants living in a shelter or on the street versus living alone after accounting for multiple predictors (OR=1.67; 95% CI: 1.19-2.37) [33].

Symptom delays are common. Early signs such as oral ulcers, hoarseness, dysphagia, or jaw pain are frequently dismissed or untreated due to a lack of dental and otolaryngology (ENT) access. PEH are far more likely to present with locally advanced or metastatic head and neck cancers, often requiring disfiguring surgery, intensive chemoradiation, or palliative care [34]. Uninsured status was associated with 21.7% vs. 9.6% more advanced disease in head and neck squamous cell carcinoma, with PEH having a four-fold higher rate of advanced-stage presentation (8.5% vs. 2.1%) [35]. Limited access to dental care is a critical upstream driver, positioning oral cancer screening and dental integration as high-impact intervention points.

Cervical Cancer

Cervical cancer remains one of the most preventable malignancies, yet PEH experience disproportionately high incidence and mortality [36]. The Boston Health Care for the Homeless cohort (2003-2008) found that cervical cancer cases and deaths occurred in excess among homeless women compared to Massachusetts adults [3]. Screening rates for Pap testing and HPV testing among unhoused women and gender-diverse individuals are extremely low, often due to fragmented care, trauma histories, mistrust of healthcare institutions, and logistical barriers such as transportation and follow-up. Per the Chicago Health Atlas, the screening rate of women without hysterectomy who have had a Pap test in the past three years in 2024 is 53.8% if <100% of the federal poverty level, compared to 72.3% if 400%+ the federal poverty level [37]. A 2025 systematic review found the pooled prevalence of cervical cancer screening in the last three years was approximately 59.8% (±6%) among PEH specifically, consistently below World Health Organization guidelines [38]. Even for those women who had been screened before, Kohler et al. call for trauma-informed care among women experiencing homelessness, as many experienced anticipated anxiety and re-traumatization that pushed them to delay or refuse future Pap smears [39]. The prevalence of high-risk HPV infection is elevated among PEH, driven by reduced vaccination coverage, higher rates of immunocompromising conditions (including HIV), and limited access to reproductive healthcare [13,40]. Consequently, cervical cancer in this population is frequently diagnosed at advanced stages, requiring more aggressive treatment and associated with poorer outcomes. A New York City study found 76% of homeless women self-reported having undergone cervical screening, but many (65%) did not know their results or did not receive follow-up on abnormal results, suggesting that women with low socioeconomic status may substantially over-report screening [40]. Mobile and community-based screening models have been identified as particularly effective for improving cervical cancer detection in marginalized populations [14]. 

Liver Cancer (Hepatocellular Carcinoma)

HCC is significantly overrepresented among PEH, largely driven by the high prevalence of chronic hepatitis C virus (HCV) and hepatitis B virus (HBV) infection, as well as heavy alcohol use [41]. PEH has some of the highest documented rates of untreated HCV in the United States, reflecting barriers to diagnosis, linkage to care, and completion of antiviral therapy. A Boston study (2003-2017) found that liver cancer was the second leading cause of liver-related death among homeless-experienced adults, with a standardized mortality ratio of 2.4 compared to the Massachusetts population [42].

Although direct-acting antiviral (DAA) therapies for HCV are highly effective and well tolerated, PEH face substantial obstacles to accessing these treatments, including insurance authorization hurdles, unstable housing, and limited specialty care access [43]. The London mobile unit (van) study found 26% HCV antibody positivity and 17.4% viremia among 940 PEH patients, of whom 56.2% were street-based. Using the mobile van to foster adherence, overall treatment initiation was 70.4%, and sustained virologic response (SVR) was 72.8%, compared with street-based homeless populations not linked to harm reduction services, who are less likely to initiate HCV treatment [44]. Without adherence, progression to cirrhosis and HCC is more common, and surveillance for liver cancer is rarely performed. Liver cancer thus exemplifies how treatable infectious disease inequities translate directly into preventable cancer deaths.

Colorectal and Breast Cancer

Screen-detectable cancers such as colorectal (CRC) and breast cancer also demonstrate disproportionate late-stage presentation among PEH. Screening rates for fecal immunochemical testing (FIT), colonoscopy, and mammography are substantially lower than national averages [45]. A New York City study (2010-2012) found only 19.7% of homeless patients at shelter-based clinics had undergone colorectal cancer screening compared to 41.3% of domiciled patients (p<0.001) [46]. Homeless patients received equal provider counseling but were more likely to decline screening. Structural barriers, including a lack of mailing addresses for FIT kits, difficulty completing bowel preparation for colonoscopy without bathroom access, and limited access to imaging facilities, severely constrain participation in standard screening pathways. Per the Chicago Health Atlas, the 2024 colorectal screening rate of adults aged 45 years or older who have had a colonoscopy or sigmoidoscopy in the past five years or a blood stool test in the past year is 54.9% vs. 81% based on the federal poverty level of <100% and 400%+, respectively [47]. The percent of women ages 45 or older having a mammogram in the past two years is 69.4% vs. 90.1% based on the federal poverty level of <100% and 400%+, respectively [48]. Coughlin noted that low socioeconomic status tends to be associated with poorer survival in colorectal cancer and later stage at diagnosis, and poorer survival in breast cancer [49,50].

When colorectal and breast cancers are diagnosed in PEH, they are more likely to be detected at regional or distant stages, resulting in lower survival and higher treatment-related morbidity [16,17,19,51]. A San Francisco public hospital study (2011-2021) found unhoused and formerly unhoused patients were more commonly diagnosed with stage 4 disease (28% and 27%, respectively, vs. 22% of housed patients). After adjusting for demographic and clinical characteristics, unhoused patients with stage 0-3 disease had a 50% increased hazard of death (aHR 1.5, 95% CI 1.1-1.9; p<0.004) [30]. These cancers represent key opportunities for mobile, low-barrier screening strategies, such as on-site FIT distribution, mobile mammography partnerships, and patient navigation models tailored to individuals without stable housing. A systematic review and meta-analysis found mailed outreach with enclosed FIT significantly increased CRC screening in low-income populations (RR 2.20, 95% CI 1.74-2.78), as did patient navigation (RR 1.62, 95% CI 1.29-2.02) [52].

In a colorectal screening study comparing three different, evidence-based reminder mechanisms at three federally qualified health clinics (FQHCs) in Chicago, Watson et al. noted an increase from a baseline screening rate of 13% to an improved rate of 20%, but the intervention was ethnically sensitive as Latinos preferred provider-delivered education, Black participants preferred lay navigation, and mixed-ethnic clinics preferred mailed birthday cards [15]. This difference highlights the importance of understanding clinical context, ethnic sensitivity, and locality when selecting which evidence-based interventions to deploy for screening.

In 2018, the University of Illinois Cancer Center and Mile Square Health Centers (MSHC) FQHC created an integrated breast cancer screening and navigation program known as the Mile Square Accessible Mammogram Outreach and Engagement (Mi-MAMO) program to tackle breast cancer disparities in under-resourced communities, noting that Chicago has the seventh highest racial disparity in breast cancer mortality (40%) in the United States [16]. They concluded that women in the program had higher cancer detection rates than national averages, that FQHCs can perform at high-quality standards, and that an integrated framework was feasible when coupling community outreach with patient navigation.

Skin Cancer

Skin cancers, including squamous cell carcinoma and melanoma, are more prevalent among unsheltered individuals due to chronic ultraviolet (UV) exposure, limited access to sun protection, and delayed evaluation of suspicious lesions. A Danish nationwide cohort study (1999-2018) of over 5 million individuals found that PEH had a 2.31-times higher incidence rate of any diagnosed skin condition compared to housed individuals. Notably, PEH had a lower occurrence of skin cancer diagnosis (aIRR 0.76, 95% CI 0.71-0.82), with only 2.8% of PEH having a skin neoplasm diagnosis vs. 5.1% of housed individuals by the end of follow-up, likely reflecting underdiagnosis rather than lower risk [53]. Chronic wounds, scars, and burn injuries may also undergo malignant transformation [54].

Across cancer types, compounded risk arises from chronic infectious diseases, repeated physical trauma, and prolonged inflammatory states, all of which are more common in homeless populations. These overlapping exposures reinforce the need for integrated, trauma-informed, and mobile healthcare approaches that address both cancer prevention and its upstream determinants.

Prostate Cancer

PEH have lower prostate cancer screening rates and generally worse cancer-related outcomes than housed populations [18]. In a study of homeless versus housed men eligible for prostate-specific antigen PSA screening in a large metropolitan health system in Cleveland from 2014 to 2021, the PSA screening rate was 13% for PEH vs. 34% for housed individuals. Half of the patients were Black. PEH were less likely to have a primary care provider (PCP) (58% vs. 81%, p<0.001). Among PEH, having Medicaid/insurance, a PCP, or being employed increased the likelihood of screening on univariate analysis. Access to the healthcare system appeared to be the largest barrier.

Barriers across the cancer care continuum 

PEH in Chicago faces a range of barriers across the cancer care continuum, ranging from prevention and screening obstacles to diagnostic challenges, as well as treatment and survivorship impediments. Forty percent of PEH are unsheltered, meaning the only way to reach them is directly on the streets [55].

Prevention and Screening Barriers

Stable primary care serves a key role in effective cancer prevention; however, the majority of unhoused individuals are less likely to have access to consistent healthcare [56]. As a result, screening rates remain substantially reduced and suffer from a lack of organized outreach efforts [10]. A Los Angeles study examining barriers to colon cancer screening among PEH found that fewer than half had health insurance or a PCP, and those who did were more likely to complete screening [57]. A separate study found that having a PCP was associated with 2.54-fold higher odds of undergoing PSA testing [18].

There is a clear need to implement cancer screening in nontraditional care settings. Because unhoused individuals often access healthcare irregularly and outside conventional clinics, prevention and awareness efforts must be delivered in shelters and on the streets where they temporarily reside. People from low socioeconomic status neighborhoods are open to direct engagement if done thoughtfully. The University of Chicago Comprehensive Cancer Center's COMPASS study demonstrated the feasibility of direct community engagement in predominantly Black, low-income Chicago neighborhoods, achieving 50%-80% favorable response rates for biospecimen collection using door-to-door recruitment with ethnically concordant interviewers [9]. Similarly, the Mi-MAMO program at MSHCs in Chicago increased breast cancer screening from 103 to 567 women annually through patient navigation, with 95.5% of participants being racial/ethnic minorities and 63.9% uninsured [16].

Diagnostic Barriers

Even when cancer screening occurs, a critical determinant of success is follow-up after abnormal screening results. Since many unhoused persons are peripatetic and lack a fixed mailing address, consistent outreach is nearly impossible, yet it is essential for a successful screening program. PEH do not have consistent access to cellular devices or internet services, both of which are recognized social determinants that influence healthcare [58]. Mobile phone ownership among PEH ranges from only 44% to 62%, and unsheltered individuals face additional challenges, including the inability to charge phones and a lack of mobile data [59]. As a result, even when healthcare is available, unhoused individuals may never receive further instructions, appointment reminders via calls or texts, or receipt of results.

It is also important to note that diagnostic processes require multiple visits with scheduled delays in between. These long waiting periods affect unhoused individuals negatively, as maintaining appointments over extended periods poses a challenge due to their unstable circumstances. Continuous delays in access to treatment increase the likelihood that cancer progresses in these individuals [12].

While Chicago-specific data on diagnostic follow-up among PEH are lacking, national studies demonstrate significant barriers. A multi-site study found that only 68% of patients with abnormal FOBT/FIT results received timely colonoscopy follow-up, with considerable variability across healthcare systems [60]. Among PEH specifically, a Veterans Affairs (VA) study found a 12% lower rate of diagnostic colonoscopy completion following positive stool-based tests compared to housed veterans [61]. Care fragmentation across multiple organizations and siloed health information systems further complicates follow-up, requiring labor-intensive manual workarounds even in well-established screening programs.

Treatment Barriers

Proper cancer treatment requires timely, recurring healthcare visits. Radiation therapy and chemotherapy, two major forms of cancer treatment, depend on daily to weekly treatment over multiple consecutive weeks with precise scheduling [17]. Homeless individuals often miss appointments due to unpredictable circumstances and limited resources. According to the 2023 Chicago Department of Public Health (CDPH) report on improving the health of PEH, a lack of consistent care can be attributed to limited transportation barriers [62]. Moreover, distant locations become inaccessible because some unhoused people are unwilling to move from their current locations to seek the care they need elsewhere in the city, which further worsens outcomes.

Housing instability also impairs the ability to safely store medications and treatment supplies, which are crucial since many cancer treatments require proper refrigeration and storage. Furthermore, environmental stability and cleanliness are vital for following proper medical procedures such as wound care and symptom management during cancer treatment [62].

Post-treatment recovery (respite care) requires the patient to rest, monitor symptoms, and prevent possible infections, especially if immunocompromised due to chemotherapy. PEHs in unsafe environments face a higher likelihood of complications, treatment termination, and hospital readmission [62]. They may not be offered the same level of services without at-home nursing care to help. Homeless people are less able to follow post-treatment instructions than permanent residents with unclear discharge instructions from acute care facilities.

Insurance instability is a common theme, especially Medicaid coverage interruptions, which can terminate cancer treatment suddenly. Medicaid churn is identified as a prominent barrier to repeated care for homeless people, even during treatment [62].

Another contributing factor to barriers to cancer treatment is the prioritization of survival needs by homeless individuals. Immediate needs such as food, shelter, and safety take precedence over traveling to medical appointments. According to the CDPH, a prominent obstacle to treatment among unhoused individuals is the reality that survival needs are often prioritized over medical issues, which may be viewed as secondary to more immediate concerns, even in the presence of serious illness [12,62].

Survivorship Barriers

Consistent monitoring is stressed by the CDC for cancer survivors, but as mentioned, PEH are often unable to follow up due to communication issues (phone-related challenges) and transportation barriers [58,62]. Furthermore, the inability of unhoused individuals to maintain long-term treatment schedules presents a challenge, as survivorship care includes laboratory testing, specialist visits, and imaging to monitor for recurrence. The living conditions of homeless individuals make strict adherence to long-term care plans very difficult, thereby increasing the possibility of cancer recurrence going undetected [13,35].

The structure of existing survivorship programs is not tailored to the PEH population. Traditional survivorship programs are designed for housed individuals with reliable access to care, resulting in persistent disparities in long-term outcomes that affect mortality rates [18,56].

Existing cancer-related resources for homeless individuals in Chicago

This section provides an overview of resources, based on publicly available descriptions of health programs and established models of care, with an attempt at direct verification at the organizational level. Patient access is largely mediated through centralized phone lines, voicemail systems, limited clinic hours, referral coordinators, or online forms, mechanisms that are often difficult for PEH to use reliably. Many programs require navigating multiple steps (e.g., calling a main line, requesting a specific navigator, or being routed through care coordination), and several explicitly note variable hours, appointment-only models, or outreach that is not consistently available. Geographic dispersion across the city and suburban Cook County further compounds these challenges, particularly for individuals with limited transportation, unstable communication access, or competing priorities related to survival needs. The primary barrier to cancer screening for unhoused populations is not simply the absence of services, but the complexity, indirectness, and logistical burden required to reach them.

Federally Qualified Health Centers and Homeless Health Programs

It is important to recognize that Chicago has comprehensive health service delivery facilities through federally qualified health centers (FQHCs) and programs serving PEH. These facilities include Heartland Health Alliance, MSHC, Erie Family Health Center, Lawndale Christian Health Center, and the Inner-City Muslim Action Network (IMAN), among others.

These entities play a critical role in early healthcare prevention and detection because they may serve as a first point of access to the healthcare system for many homeless individuals. While few engage in cancer-specific programs, they provide access to basic screening services (breast, cervical, and colorectal referrals), risk-factor counseling, and referrals to cancer and oncology safety-net services. Nonetheless, because of restricted resource availability and other primary care priorities, cancer treatment services do not appear to be readily available or incorporated.

Street Medicine Teams

Street medicine services provide healthcare in unconventional settings by engaging patients who are disconnected from healthcare services provided at healthcare facilities. In Chicago, the main health providers are Cook County Health Street Medicine, University of Illinois (UI) Health Street Outreach, Chicago Street Medicine, Loyola Street Medicine, The Night Ministry, and Chi Care Medical.

These teams work to address acute and chronic illnesses such as respiratory disease, hypertension, diabetes, wound care, mental illness, substance abuse, and harm reduction. However, they are an underutilized resource for cancer prevention and intervention through a mobile platform. Oncology is not currently a major focus, as the problem is considered too complex to address. This stems from limited access to imaging and biopsies, challenges with treatment follow-through, and reliance on multiple healthcare systems for different components of cancer care.

Safety-Net Oncology Systems

The safety-net hospitals in Chicago represent the primary centers of cancer care for the uninsured. Cook County Health is the major safety-net provider for patients with cancer and offers a wide array of comprehensive cancer care services, including imaging, pathology, surgery, chemotherapy, and radiation therapy. Other resources include the University of Illinois Health Cancer Center and community-based programs affiliated with Rush University Medical Center, Sinai Chicago, and Northwestern Medicine. All of these systems offer high-quality cancer treatment programs. However, PEH encounters a range of challenges stemming from a lack of insurance and the complicated process of applying for compassionate care.

Medical Respite and Housing Programs

Medical respite care and supportive housing are key elements in cancer care adherence and survival. Nonprofit organizations such as The Boulevard, Housing Forward, Franciscan Outreach, and The Salvation Army Medical Respite offer ill individuals a place to recover in shelters and temporary housing under continuous observation.

These programs, although not cancer-specialized, are highly important for post-operative care, including infection prevention, chemotherapy tolerance, and symptom control. However, limited bed capacity and, more significantly, the lack of cancer-specialized temporary respite facilities remain major deficiencies in the healthcare system. Even very short-term housing to allow colonoscopy preparation with bathroom access is needed. Partnerships between healthcare systems, shelters, and housing advocacy organizations can facilitate short-term placements that prioritize proximity to treatment centers and access to supportive services. These collaborations reduce emergency department utilization, improve continuity of care, and address housing instability as a core determinant of cancer outcomes.

Mobile Screening Programs

Mobile screening programs and community-based screening opportunities are poised to help fill the gap in early detection. However, these programs frequently lack complete linkage between diagnostic evaluation and treatment. Effective follow-up requires robust electronic medical records (EMRs) with current contact information to communicate results directly to patients rather than relying on their return to the clinic. To date, cancer prevention has not been a central focus of most mobile medical programs, not because of limited importance, but because of the perceived complexity of implementation within a fragmented healthcare system. Programs like The Night Ministry and the Chi Care mobile medical van should embed cancer screening within broader primary care and harm-reduction services. Delivering screenings in conjunction with wound care, infectious disease evaluation, chronic disease management, and social support reframes cancer prevention as a routine component of care, thereby improving screening participation and facilitating sustained engagement.

Table 1 illustrates the broad yet fragmented network of cancer screening and oncology-related services available, where access is often hindered by complex referral pathways, limited outreach availability, transportation barriers, and reliance on phone- or appointment-based systems, underscoring the need for low-barrier, mobile, and embedded cancer screening models.

Major gaps in Chicago's current system

Despite its strong network of safety-net hospitals, FQHC, street medicine teams, and medical respite programs, there are still some glaring structural flaws within the system of care for PEH with cancer in Chicago. These issues exist throughout the cancer care continuum.

First, it is important to recognize that no citywide cancer screening strategy specifically targets PEH. Screening efforts remain fragmented and nonuniform across facilities, shelters, and mobile programs, resulting in a process that is uncoordinated and dependent on the site of care.

Second, Chicago lacks dedicated patient navigation services for individuals with cancer who are unstably housed. While safety-net programs provide basic care coordination, navigation services specifically tailored to the needs of unhoused patients, such as communication barriers, transportation limitations, and competing survival priorities, remain insufficiently developed and implemented. In a systematic review, Dommaraju et al. demonstrated that patient navigation was associated with substantial improvements in screening outcomes, including one study reporting 8.51-fold higher odds of completing lung cancer screening [14]. Another important aspect is transportation; the lack of travel options greatly hinders people's access to imaging services, cancer appointments, and treatment centers. This is especially true when treatment involves frequent visits over an extended period. Current transportation assistance programs available to those in need do not meet the demands of cancer treatment. Free public transportation may be difficult to use when individuals are sufficiently ill. As a result, major treatment may require hospitalization rather than outpatient services, driving up costs.

Dental and medical care systems remain largely disconnected, with most dental services delivered through outpatient clinics. Many homeless healthcare facilities lack the capacity to provide comprehensive dental services, including pre-radiation dental clearance, despite well-established links between dental disease, tobacco use, and head and neck cancers.

Furthermore, capacity within medical respite programs is inadequate for individuals undergoing cancer treatment or recovering from surgery, chemotherapy, or radiation therapy. Although respite care programs exist, they cannot logistically support the level of recovery needed by many cancer patients; the number of available beds does not meet demand. Expansion of respite capacity specifically for oncology patients represents a high-impact, scalable investment.

Data infrastructure represents a critical weakness. Chicago lacks a shared EMR accessible across systems to monitor outcomes among PEH, with most institutional records remaining siloed and inaccessible to partner organizations. Moreover, the absence of a homeless-specific cancer registry complicates efforts to evaluate clinical outcomes. Shelter-hospital data-sharing agreements can improve care coordination while preserving patient privacy. Dedicated funding for outcomes research and staging data collection is critical to inform policy, allocate resources, and evaluate the effectiveness of interventions.

Finally, street medicine programs lack standardized protocols for symptom-based prioritization, screening, referral, and cancer risk assessment. Cancer prevention and early detection are not routinely integrated into street-based care, despite these teams' unique access to hard-to-reach populations and the substantial cancer burden among PEH.

The need for an expanded role of street medicine and mobile clinics in oncology

Mobile healthcare clinics represent one of the most effective and scalable strategies for delivering a wide range of services to PEH [63]. Typical services include primary care, behavioral health, and social services [64]. However, street medicine and mobile clinics are a relatively recent phenomenon, with general guidelines for working with PEH published in 2004 and international primary care guidelines published by the Street Medicine Institute in 2018 [64]. By eliminating transportation barriers, reducing mistrust of traditional healthcare institutions, and embedding care within familiar community settings, mobile clinics serve as critical entry points into the cancer care continuum. Forty percent of PEH are unsheltered, meaning the only way to reach them is directly on the streets [55].

Given that fixing the overall healthcare system is beyond the scope of any single organization or this review, the immediate solution appears to be offering direct screening through a mobile platform based on recommendations in the medical literature. Decker et al. recommend improving access to colorectal cancer screening by “embracing innovative and patient-centered methods, including roving street medicine teams, drop-in clinics, and embedding care within shelter systems” [65]. We propose a detailed implementation strategy for each cancer screening modality using mobile programs that reach PEH directly on the streets or in shelters through pop-up clinics.

Lung Cancer Screening

Mobile programs should focus on smoking cessation, risk assessment, shared decision-making, and navigation to LDCT facilities rather than attempting to provide LDCT imaging directly [1]. The navigator provides basic education on the benefits and risks of lung cancer screening, but an individualized decision about the appropriateness of screening is ultimately made by the patient and the mobile care provider. Risk assessment includes documenting pack-year smoking history, environmental factors (asbestos, silica, and diesel fumes), radon exposure, family history, and history of COPD or pulmonary fibrosis [66]. All patients who currently use tobacco should be offered support and resources to help them reduce or quit smoking (smoking cessation), including nicotine patches. Studies in homeless populations show 70% quit attempt rates and high acceptability of nicotine replacement therapy when delivered in the shelter setting [66]. The use of decision aids can help shared decision-making in regard to benefits (20% lung cancer mortality reduction) and harms (false positives and incidental findings). The navigation should assist with scheduling at imaging facilities, provide transportation assistance, send appointment reminders, and coordinate with radiology departments for results [67]. Ideally, follow-up coordination would establish protocols for tracking lung imaging reporting and data system category 3 (Lung-RADS 3) and four results with an EMR connected to the screening facility.

Colorectal Cancer Screening

FIT testing is ideal for mobile programs as it requires only stool collection with no dietary restrictions or bowel preparation [68]. FIT kits can be distributed directly during mobile clinic encounters or at shelter-based visits along with clear, low-literacy instructions in multiple languages. Due to the lack of private bathroom access, a program can consider partnering with shelters to designate collection facilities or establish multiple return options, including mobile pick-up, shelter-based collection boxes, or pre-paid mailing envelopes. Even with screening, only 25% of PEH with positive FIT tests (compared to about 50% in the general population) complete colonoscopy [69]. Most programs fail because of inadequate follow-up infrastructure. For positive FIT results, patient navigation must include insurance assistance, gastroenterology referral coordination, bowel preparation education with attention to bathroom access, transportation arrangements, accompaniment to procedures, and aggressive follow-up to ensure completion within nine months per NCCN guidelines [70]. A FIT-first model would reduce the colonoscopy burden on healthcare systems.

Cervical Cancer Screening

HPV self-collection kits represent one of the strongest screening modalities for mobile programs following recent FDA approval (2024-2025) and endorsement by the American Cancer Society as an acceptable alternative to clinician-collected Pap smear screening [71]. Mailed self-collection kits increased screening participation 2.5-fold (41%-47% vs. 16%) in safety-net settings, with over 80% of screened individuals choosing self-collection over clinic-based screening [71]. Self-administration enhances trauma-informed care by increasing patient control and comfort while reducing potential triggers in women who have experienced sexual trauma [72]. Kits should provide clear visual instructions in multiple languages, with consideration for demonstration models or video instructions accessible via QR codes. Street medicine programs can establish prepaid mailing systems or drop-off locations at mobile clinic sites and partner shelters. HPV vaccination can easily be offered in mobile settings to eligible individuals (ages 9-26 years, with shared decision-making for ages 27-45). As with colonoscopy, the program will need to coordinate colposcopy referrals for HPV-positive results.

Breast Cancer Screening

Mobile mammography requires significant infrastructure but can be implemented through partnerships between community agencies and imaging facilities. Rush MD Anderson and Roseland Community Hospital operate mobile mammography units in Chicago. Mobile street medicine programs could coordinate group appointments for multiple PEH to attend together, reducing isolation and no-show rates. Trusted shelter or mobile clinic staff could accompany individuals to mammography appointments. Alternatively, programs could arrange group transportation to non-mobile imaging facilities such as Mi-MAMO [16].

Prostate Cancer Screening

PSA testing is feasible in mobile settings as it requires only a blood draw. Programs could utilize point-of-care PSA testing, if available, or coordinate with laboratory services for blood collection and processing. Shared decision-making is necessary because PSA screening recommendations are nuanced and require individualized discussion of benefits and harms, particularly for men ages 55-69 years. A program would need referral protocols for urology and radiation oncology services [18].

Head and Neck Cancer Screening

Physical examination screening is highly feasible in mobile settings and requires minimal equipment. Providers can perform an oral cavity examination with visual inspection and palpation of the oral mucosa, tongue, floor of the mouth, and oropharynx, along with a neck examination, including palpation for lymphadenopathy. Risk assessment would document tobacco and alcohol use history. HPV vaccination can be provided, with a two-dose series (6-12 months apart) for those initiating vaccination before age 15, while a three-dose series is recommended for those initiating vaccination between ages 15 and 45. The program should establish a clear referral protocol to otolaryngology for suspicious lesions and to dental clinics for general oral healthcare.

Skin Cancer Screening

Full-body skin examination can be performed in mobile settings with adequate privacy. This can only be done if trust is established with culturally and trauma-sensitive providers. A systematic examination of all skin surfaces can be performed visually, with dermoscopy or smartphone-based imaging (photography) used for suspicious lesions requiring dermatology consultation. Providers can offer education on sun exposure risks, particularly for PEH with significant outdoor exposure.

Liver Cancer Prevention

Mobile units can administer the HBV vaccine series to unvaccinated individuals, as HBV causes liver cancer. HCV screening can be performed with point-of-care rapid HCV antibody testing, followed by linkage to confirmatory RNA testing and treatment. DAAs cure HCV and prevent progression to cirrhosis and HCC. Mobile units can assist with scheduled visits to encourage adherence to treatment and improve treatment completion [44].

Patient Navigation and Additional Support Network

A core need within each mobile street medicine program is patient navigation, which provides multidimensional, personalized assistance across the cancer care continuum. Patient navigation is the most evidence-based intervention for improving cancer screening among PEH, with demonstrated 4.7-fold increases in lung cancer screening completion and 8.51-fold higher odds of screening completion in randomized trials [3,14]. Navigators should be college graduates who undergo focused education and training in patient navigation, cancer screening, and tobacco counseling. Ideally, they should possess cultural competence; staff with shared lived experiences or a deep understanding of homelessness are critical for trust-building [14]. They need to be trained in cancer screening guidelines, risk communication, motivational interviewing, trauma-informed care, and health systems navigation [73]. Based on the INHALE trial and systematic reviews, core navigator duties should include providing cancer-specific education tailored to language and literacy level, addressing misconceptions about cancer and screening, discussing personal risk factors and benefits of early detection, and using decision aids to support decision-making [3,14]. The navigation protocol should be structured yet flexible to adapt to individual needs, with interactions dependent on the patient’s needs.

Social workers can help with logistical coordination, such as scheduling appointments based on geographic proximity and patient preference, providing appointment reminders through multiple modalities (in-person shelter visits, phone calls, and text messages), arranging transportation assistance and travel vouchers, and coordinating with PCPs, specialists, and imaging facilities. They can assist with insurance enrollment and coverage verification, provide incentives (e.g., gift cards and transportation vouchers), address language barriers through interpreter services, and help navigate bowel preparation for colonoscopy with attention to bathroom access.

Other Considerations

A mobile program would ideally have robust follow-up and tracking systems. Program staff could obtain screening results on behalf of patients (with consent) and communicate results to patients and providers. They should document all activities using standardized checklists and arrange follow-up visits or studies when needed. Such data are needed to identify barriers, document successes, improve quality measures, and support applications for financial grants.

Communication challenges are the primary impediment to navigation success, particularly for individuals currently experiencing homelessness [8]. Each patient will require multiple contact methods, including attempts through shelter visits, shelter phones, personal cell phones, and case manager coordination. Providing phones with prepaid minutes enables appointment reminders, care coordination, and emergency communication. Outreach will need to be persistent and flexible to accommodate competing survival priorities and unpredictable daily circumstances. Partnerships with shelters could help integrate navigation with existing shelter case management systems. Telehealth kiosks within shelters can facilitate oncology follow-ups, symptom monitoring, and specialist consultations.

**Figure 2:**
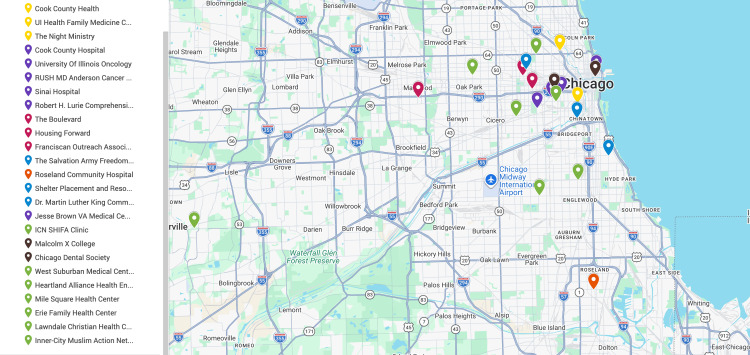
Geographic distribution of cancer screening and oncology resources serving PEH in Chicago Figure created by the authors with the use of Google My Maps (Google Limited Liability Company, Mountain View, CA, USA) PEH, people experiencing homelessness; ICN, Islamic Circle of North America; UI, University of Illinois

Cancer screening does not occur in a vacuum of other social problems. It requires integration with social services, including housing assistance referrals, food security support, mental health and substance use services, and primary care coordination (Figure 2).

Policy recommendations

Addressing cancer disparities among PEH requires coordinated citywide policy action across healthcare, housing, and public health systems. Expanding Medicaid navigation and enrollment support is a foundational step to ensure continuity of cancer screening, diagnostic workup, and treatment coverage, particularly for individuals with unstable housing and frequent insurance disruptions.

Sustainable public funding for shelter-based and community-based health clinics should be prioritized to support low-barrier cancer screening, follow-up care, and patient navigation within trusted environments. Policies that incentivize hospitals and health systems to routinely screen for housing instability and integrate that information into care planning can improve early identification of high-risk patients and reduce loss to follow-up.

Municipal and state governments should also increase dedicated funding for medical respite programs and mobile cancer screening initiatives, recognizing these services as essential components of the cancer care continuum rather than optional social supports. Finally, city-level integration of cancer prevention, screening, and treatment into homelessness response strategies, including formal partnerships among public health departments, hospitals, shelters, and outreach organizations, can ensure that cancer equity is embedded within broader efforts to address homelessness in Chicago.

From a funding and scalability perspective, mobile cancer prevention programs offer favorable cost-effectiveness through earlier detection. A Harvard study calculated the return on investment for mobile healthcare as 36:1 for a single Family Van operating in Boston in 2008 [74]. As the mobile van provided seven of the top 25 priority prevention services, the authors compared the projected annual emergency department costs avoided and the value of potential life-years saved from services rendered in a year (total $20.3 million) with the annual cost of the mobile program (approximately $0.57 million). At the time of publication, the van had been in operation for 16 years with broad public support due to savings of $36 for every $1 invested. Early detection through mobile screening reduces downstream costs associated with late-stage cancer treatment, emergency department utilization, and avoidable hospitalizations. Philanthropic and public funding can support vehicle acquisition and maintenance (e.g., Mobile Care Chicago), portable diagnostic equipment, staffing (including clinicians, navigators, and outreach workers), and partnerships with imaging centers and oncology providers.

Ultimately, mobile care clinics represent a pragmatic, equity-centered solution to cancer disparities among PEH. By bringing prevention, screening, navigation, and trust-based care directly into the community, mobile models transform cancer care from a system that passively waits for patients to present into one that actively reaches those most at risk.

Limitations

This study has several limitations. As a narrative scoping review, it did not employ a formal systematic review methodology, PRISMA-guided study selection process, or validated risk-of-bias assessment, which limits the ability to draw definitive causal conclusions from the synthesized literature. The evidence base for cancer-specific outcomes among PEH remains sparse overall, and this limitation was particularly pronounced at the regional level - only two Chicago-specific studies and three Midwest-based studies directly examined cancer screening among homeless populations. This geographic gap necessitated reliance on national-level data and extrapolation from other urban settings, which may not fully capture the unique healthcare infrastructure, shelter systems, and demographic composition of Chicago's homeless population. Additionally, because cancer-specific data stratified by housing status are rarely collected in cancer registries or hospital discharge databases, some analyses relied on poverty level as a surrogate marker for homelessness, which likely underestimates the true burden of cancer disparities in this population. Publication bias may also affect the findings, as studies demonstrating positive outcomes from navigation interventions may be more likely to be published than studies with null results. Finally, the heterogeneity of study designs, populations, and outcome measures across included sources precluded quantitative synthesis or meta-analysis.

Future directions

Future research should prioritize the generation of Chicago-specific primary data to address the gaps identified in this review. A critical next step is the administration of structured questionnaires at homeless shelters, transitional housing programs, and street medicine outreach sites across the city to directly assess cancer knowledge, screening history, perceived barriers to care, and healthcare preferences among PEH. Such surveys should be designed with input from community stakeholders and individuals with lived experience of homelessness to ensure cultural relevance and minimize response bias. Prospective cohort studies tracking cancer screening uptake, stage at diagnosis, treatment completion, and survival outcomes among PEH engaged in mobile oncology outreach programs would provide the strongest evidence for program effectiveness. Integration of housing status as a standardized variable in cancer registries and electronic health records would enable more accurate epidemiologic surveillance and reduce reliance on poverty-based surrogates. Implementation science frameworks should be applied to evaluate the feasibility, acceptability, and sustainability of mobile oncology models in Chicago's distinct geographic and demographic landscape, particularly on the South and West Sides, where cancer disparities and homelessness are most concentrated. Finally, multi-city collaborative studies across Midwestern urban centers could help determine whether findings from coastal cities are generalizable to the region's unique healthcare and social service ecosystems.

## Conclusions

Cancer among PEH is not an inevitable consequence of poverty or instability; it is a preventable and solvable health inequity driven by delayed diagnosis, fragmented care, and systemic barriers. These outcomes reflect failures in access and coordination, not individual choice or nonadherence. Chicago is uniquely positioned to lead national efforts to close this gap. With its robust safety-net hospital systems, active public health infrastructure, and deeply rooted community organizations, the city has the capacity to reimagine cancer care for those most often excluded from it. Targeted investments in transportation access, patient navigation, dental and ENT services, medical respite, and mobile screening programs can dramatically improve survival, reduce suffering, and restore dignity for individuals experiencing homelessness.

Street medicine programs, including mobile medical van services and pop-up clinics at shelters, represent a newer model for delivering care directly within communities through trusted, low-barrier, relationship-centered outreach connected to larger healthcare systems. Improved integration of screening, navigation, and follow-up within mobile primary care demonstrates that equity-focused cancer care is attainable. Continued expansion of and investment in these models could improve access to cancer care for the most vulnerable by removing the earliest barrier to access, meeting unhoused individuals where they are rather than waiting for them to present for care.

## References

[1] (2025). Hidden homelessness in the U.S.: why Congress must change HUD's definition of homelessness to align with other federal agencies. https://schoolhouseconnection.org/article/hidden-homelessness-in-the-u-s-why-congress-must-change-huds-definition-of-homelessness-to-align-with-other-federal-agencies.

[2] (2026). Chicago Sun-Times: Chicago's homelessness surged in 2024, as major U.S. cities bore the brunt of a national trend. https://chicagohomeless.org/chicago-sun-times-chicagos-homelessness-surged-in-2024-as-major-u-s-cities-bore-the-brunt-of-a-national-trend/.

[3] Baggett TP, Chang Y, Porneala BC, Bharel M, Singer DE, Rigotti NA (2015). Disparities in cancer incidence, stage, and mortality at Boston health care for the homeless program. Am J Prev Med.

[4] (2026). Poverty rate. https://chicagohealthatlas.org/indicators/POV?topic=poverty-rate.

[5] Caine P (2024). Illinois taking steps to reduce high rates of homelessness in Black community. https://news.wttw.com/2024/05/01/illinois-taking-steps-reduce-high-rates-homelessness-black-community.

[6] (2024). Chronic disease indicators: cancer. https://www.cdc.gov/cdi/indicator-definitions/cancer.html.

[7] (2026). Common cancer sites: cancer stat facts. https://seer.cancer.gov/statfacts/html/common.html.

[8] Baggett TP, Sporn N, Barbosa Teixeira J (2025). Homelessness, patient navigation, and lung cancer screening in a health center setting: a subgroup analysis of a randomized clinical trial. JAMA Netw Open.

[9] Odunsi K (2023). Perspectives on disparities and equity in cancer outcomes: a call to action. Acad Med.

[10] (2025). Chicago Alliance to End Homelessness, Loyola University Chicago Center for Urban Research and learning: homeless over 50: the graying of Chicago's homeless population. https://nhchc.org/wp-content/uploads/2019/08/homeless_Over_50_Report.pdf.

[11] Fan Q, Nogueira L, Yabroff KR, Hussaini SM, Pollack CE (2022). Housing and cancer care and outcomes: a systematic review. J Natl Cancer Inst.

[12] Drescher NR, Oladeru OT (2023). Cancer screening, treatment, and outcomes in persons experiencing homelessness: shifting the lens to an understudied population. JCO Oncol Pract.

[13] Asgary R (2024). Cancer care and treatment during homelessness. Lancet Oncol.

[14] Dommaraju SR, Koch RM, Mayo A, Tevaarwerk A (2025). Innovative interventions that promote access to cancer care among people experiencing homelessness and lessons learned: a systematic review. J Health Care Poor Underserved.

[15] Watson KS, Tossas KY, San Miguel Y (2023). MI-CARE: comparing three evidence-based interventions to promote colorectal cancer screening among ethnic minorities within three different clinical contexts. Int J Environ Res Public Health.

[16] Henderson V, Tossas-Milligan K, Martinez E (2020). Implementation of an integrated framework for a breast cancer screening and navigation program for women from underresourced communities. Cancer.

[17] Kilic SS, Mayo ZS, Weleff J (2023). Cancer diagnoses and use of radiation therapy among persons experiencing homelessness. Int J Radiat Oncol Biol Phys.

[18] Mayo ZS, Kilic SS, Weleff J (2022). Prostate cancer screening disparities in persons experiencing homelessness. JCO Oncol Pract.

[19] Holowatyj AN, Heath EI, Pappas LM (2019). The epidemiology of cancer among homeless adults in metropolitan Detroit. JNCI Cancer Spectr.

[20] Baggett TP, Tobey ML, Rigotti NA (2013). Tobacco use among homeless people-addressing the neglected addiction. N Engl J Med.

[21] Gragnani L, Monti M, De Giorgi I, Zignego AL (2025). The key importance of screening underprivileged people in order to achieve global hepatitis virus elimination targets. Viruses.

[22] Vanderpool RC, Stradtman LR, Brandt HM (2019). Policy opportunities to increase HPV vaccination in rural communities. Hum Vaccin Immunother.

[23] Freitas DJ, Kaplan LM, Tieu L, Ponath C, Guzman D, Kushel M (2019). Oral health and access to dental care among older homeless adults: results from the HOPE HOME study. J Public Health Dent.

[24] Solmi M, Firth J, Miola A (2020). Disparities in cancer screening in people with mental illness across the world versus the general population: prevalence and comparative meta-analysis including 4 717 839 people. Lancet Psychiatry.

[25] Chase B, Judge P (2026). Pollution hits Chicago’s West, South Sides hardest. https://illinoisanswers.org/2018/10/25/interactive-map-pollution-hits-chicagos-west-south-sides-hardest/.

[26] Campbell DJ, O'Neill BG, Gibson K, Thurston WE (2015). Primary healthcare needs and barriers to care among Calgary's homeless populations. BMC Fam Pract.

[27] eClinicalMedicine eClinicalMedicine (2023). Equitable health care for people experiencing homelessness. eClinicalMedicine.

[28] Coughlin SS (2021). Social determinants of health and cancer survivorship. J Environ Health Sci.

[29] Heilig D, Szabó Á, Fadgyas-Freyler P, Simon J (2025). The impact of homelessness on lung cancer survival and healthcare utilization in the Hungarian universal healthcare system. Cancers (Basel).

[30] Decker H, Colom S, Evans JL (2024). Association of housing status and cancer diagnosis, care coordination and outcomes in a public hospital: a retrospective cohort study. BMJ Open.

[31] Döbrossy L (2005). Epidemiology of head and neck cancer: magnitude of the problem. Cancer Metastasis Rev.

[32] Yusuf H, Golkari A, Kaddour S (2023). Oral health of people experiencing homelessness in London: a mixed methods study. BMC Public Health.

[33] Badri P, Lai H, Ganatra S, Baracos V, Amin M (2022). Factors associated with oral cancerous and precancerous lesions in an underserved community: a cross-sectional study. Int J Environ Res Public Health.

[34] Moore CE, Durden F (2010). Head and neck cancer screening in homeless communities: heal (health education, assessment, and leadership). J Natl Med Assoc.

[35] Victor MT, Zheng W, Park SJ, Jiang SI, Guo TW (2024). Insurance status is associated with recurrence in cutaneous head and neck squamous cell carcinoma. Otolaryngol Head Neck Surg.

[36] Rodriguez NM, Balian L, Ziolkowski R, Case X, Smith K, Tipton J (2024). Community-informed interventions to address cervical cancer disparities among people experiencing homelessness. Cancer Epidemiol Biomarkers Prev.

[37] (2026). Cervical cancer screening rate. http://chicagohealthatlas.org/indicators/HCSCVP?tab=chart.

[38] Galvin AM, Akpan IN, Garg A, Cuccaro PM, Thompson EL, Santa Maria DM (2025). Human papillomavirus-related cancer prevention among people experiencing housing instability: a systematic review. Sex Transm Dis.

[39] Kohler RE, Roncarati JS, Aguiar A, Chatterjee P, Gaeta J, Viswanath K, Henry C (2021). Trauma and cervical cancer screening among women experiencing homelessness: a call for trauma-informed care. Womens Health (Lond).

[40] Asgary R, Alcabes A, Feldman R, Garland V, Naderi R, Ogedegbe G, Sckell B (2016). Cervical cancer screening among homeless women of New York City shelters. Matern Child Health J.

[41] El-Serag HB, Kanwal F (2014). Epidemiology of hepatocellular carcinoma in the United States: where are we? Where do we go?. Hepatology.

[42] Adams LD, Dickins KA, Lewis E, Beiser ME, Baggett TP, Fine DR (2025). Liver-related mortality in homeless-experienced adults over a 16-year period. J Health Care Poor Underserved.

[43] Millman AJ, Ntiri-Reid B, Irvin R (2017). Barriers to treatment access for chronic hepatitis C virus infection: A case series. Top Antivir Med.

[44] Guerra-Veloz MF, Han K, Oakes K (2023). Results of a model of delivering hepatitis C care in a homeless metropolitan population in England. Am J Gastroenterol.

[45] Al-Assil T, Kalina C, Laird MC (2025). Sheltered yet unscreened: exploring cancer screening rates and barriers in the unhoused (homeless) population. Am J Surg.

[46] Asgary R, Garland V, Jakubowski A, Sckell B (2014). Colorectal cancer screening among the homeless population of New York City shelter-based clinics. Am J Public Health.

[47] (2026). Colorectal cancer screening rate. http://chicagohealthatlas.org/indicators/HCSCRP?topic=colorectal-cancer-screening-rate.

[48] (2026). Breast cancer screening rate. http://chicagohealthatlas.org/indicators/HCSBCP?topic=breast-cancer-screening-rate.

[49] Coughlin SS (2020). Social determinants of colorectal cancer risk, stage, and survival: a systematic review. Int J Colorectal Dis.

[50] Coughlin SS (2019). Social determinants of breast cancer risk, stage, and survival. Breast Cancer Res Treat.

[51] (2026). The hidden health crisis: mortality and morbidity among people experiencing homelessness. http://publichealth.uic.edu/news-stories/the-hidden-health-crisis-mortality-and-morbidity-among-people-experiencing-homelessness-in-illinois/.

[52] Rubin L, Okitondo C, Haines L, Ebell M (2023). Interventions to increase colorectal cancer screening adherence in low-income settings within the United States: a systematic review and meta-analysis. Prev Med.

[53] Nilsson SF, Ali Z, Laursen TM (2023). Association of homelessness and skin conditions: a Danish population-based cohort study. Br J Dermatol.

[54] Gallagher K, Talasila S, Bistline A, Krain R, Ramani L, Jones E (2024). Addressing dermatologic concerns and teledermatology in undomiciled and sheltered populations. Arch Dermatol Res.

[55] (2026). Centers for Disease Control and Prevention: about homelessness and health. https://www.cdc.gov/homelessness-and-health/about/index.html.

[56] Asgary R, Sckell B, Alcabes A, Naderi R, Ogedegbe G (2015). Perspectives of cancer and cancer screening among homeless adults of New York City shelter-based clinics: a qualitative approach. Cancer Causes Control.

[57] Burra A, Gonzales N, Jiang J (2025). Understanding barriers to colon cancer screening among individuals experiencing housing insecurity in Los Angeles. Cureus.

[58] Benda NC, Veinot TC, Sieck CJ, Ancker JS (2020). Broadband internet access is a social determinant of health!. Am J Public Health.

[59] Agustin R, Garber K, Barthold G, Gustin T (2026). Evaluating access to cellphones for telehealth among people experiencing homelessness. Telemed J E Health.

[60] McCarthy AM, Kim JJ, Beaber EF (2016). Follow-up of abnormal breast and colorectal cancer screening by race/ethnicity. Am J Prev Med.

[61] Decker H, Graham L, Titan A, Hawn M, Kushel M, Kanzaria HK, Wick E (2025). Housing status and cancer screening in US veterans. J Gen Intern Med.

[62] (2026). Report on the recommendations of the systems change collaborative to improve the health of people experiencing homelessness. https://www.chicago.gov/content/dam/city/depts/cdph/fact-sheets-reports-studies/CDPH-Systems-Change-Report_final_May2023-ax.em.pdf.

[63] Malone NC, Williams MM, Smith Fawzi MC, Bennet J, Hill C, Katz JN, Oriol NE (2020). Mobile health clinics in the United States. Int J Equity Health.

[64] Kaufman RA, Mallick M, Louis JT, Williams M, Oriol N (2024). The role of street medicine and mobile clinics for persons experiencing homelessness: a scoping review. Int J Environ Res Public Health.

[65] Decker H, Erickson C, Wick E (2025). Addressing colorectal cancer disparities in unhoused populations: a call for equitable access and compassionate care. Clin Colon Rectal Surg.

[66] Hartman-Filson M, Chen J, Lee P (2022). A community-based tobacco cessation program for individuals experiencing homelessness. Addict Behav.

[67] Baggett TP, Sporn N, Barbosa Teixeira J (2024). Patient navigation for lung cancer screening at a health care for the homeless program: a randomized clinical trial. JAMA Intern Med.

[68] Belon AP, McKenzie E, Teare G (2024). Effective strategies for fecal immunochemical tests (FIT) programs to improve colorectal cancer screening uptake among populations with limited access to the healthcare system: a rapid review. BMC Health Serv Res.

[69] Stone BK, Gates JI, Monteiro KA (2023). Social determinants of health: are colonoscopies always fit for duty?. Am J Manag Care.

[70] Thompson JH, Schneider JL, Rivelli JS (2026). Implementing a successful patient navigation program for follow-up colonoscopy: Lessons from the PRECISE study. PLoS One.

[71] Montealegre JR, Hilsenbeck SG, Bulsara S (2025). Self-collection for cervical cancer screening in a safety-net setting: the Prestis randomized clinical trial. JAMA Intern Med.

[72] Madding RA, Currier JJ, Yanit K, Hedges M, Bruegl A (2024). HPV self-collection for cervical cancer screening among survivors of sexual trauma: a qualitative study. BMC Womens Health.

[73] Nelson HD, Cantor AG, Pappas M, Blackie K, Yu Y, Fu R (2025). Patient navigation services for breast and cervical cancer screening and follow-up: a meta-analysis. JAMA Intern Med.

[74] Oriol NE, Cote PJ, Vavasis AP, Bennet J, Delorenzo D, Blanc P, Kohane I (2009). Calculating the return on investment of mobile healthcare. BMC Med.

